# Clinic- and Hospital-Based Home Care, Outpatient Parenteral Antimicrobial Therapy (OPAT) and the Evolving Clinical Responsibilities of the Pharmacist

**DOI:** 10.3390/pharmacy8040233

**Published:** 2020-12-07

**Authors:** Toni Docherty, Jennifer J. Schneider, Joyce Cooper

**Affiliations:** 1 Central Coast Local Health District, Gosford, NSW 2250, Australia; toni.docherty@uon.edu.au; 2 Faculty of Health and Medicine, University of Newcastle, Callaghan, NSW 2308, Australia; jennifer.schneider@newcastle.edu.au

**Keywords:** care, hospital based home, home infusion therapies, infectious disease medicine, pharmacist

## Abstract

Clinic- and hospital-based home care describes models of care where services commonly associated with hospital inpatient care are provided at the patient’s home or in an outpatient or community-based clinic. Hospital in the Home (HITH), also termed Hospital at Home (HaH) in parts of Europe and America, is a common and important example of this type of care. Other examples include infusion centers, skilled nursing facilities (particularly in the USA), self-administration models (including home infusion services) and administration through outpatient or community clinics. Different models of HITH care are used internationally and these encompass a wide range of services. Medication administration, particularly outpatient parenteral antimicrobial therapy (OPAT), is an important element in many of these models of care. There is a key role for pharmacists since the provision of medication is integral in this model of patient care outside the hospital setting. Data on the growing importance of HITH and OPAT as well as the administration of medications suited to clinic- and hospital-based home care, including subcutaneous and intramuscular injectables, immunoglobulins and other blood fractions, cancer chemotherapy, total parenteral nutrition, biologicals/biosimilars, vasopressors and enzymes, using differing service models, are described. The pharmacist’s role is evolving from that involved primarily with dose preparation and supply of medications. Their clinical expertise in medication management ensures that they are an integral member and leader in these models of care. Their role ensures the safe and quality use of medicines, particularly across transitions of care, with the pharmacist taking on the roles of educator and consultant to patients and health professional colleagues. Activities such as antimicrobial stewardship and ongoing monitoring of patients and outcomes is fundamental to ensure quality patient outcomes in these settings.

## 1. Introduction

Clinical care commonly associated with hospital inpatient care has been delivered in the home setting internationally for many decades. Varying terminology is used to describe this type of care. In 1953, The Canadian Journal of Public Health described a new healthcare service called ‘Hospital Home Care’. It stated that ‘the patient is considered a hospital patient, where the patient is treated at home with hospital resources’ and that ‘Hospital home-care plans are still so new that their eventual scope is not fully known’ [1]. A few years later in France, the first Hospital in the Home Service, called ‘L’Hospitalisation A Domicile’ or ‘HAD’, was introduced in Paris in 1957, and it grew to 207 services throughout the country by 2007 [2]. In Australia, this type of care, providing clinical care to patients outside of the hospital setting, most commonly in their own homes, is generally termed Hospital in the Home (HITH), with an increasing acceptance of HITH services and the Australian Department of Health encouraging this expansion [3]. A recent Australian study observed that between 2011 and 2017, HITH patient admissions grew by almost twice the rate of all hospital admissions in a group of 20 Australian principal referrer hospitals [4]. Delivery of intravenous antimicrobial therapy is an important part of many of these clinic and home-based services, initially utilized by patients with cystic fibrosis receiving outpatient intravenous antibiotic therapy (OPAT) to assist in the management of their condition in their home environment since the 1970s [5].

A key element in these models of care is the provision of medicines, traditionally provided in an inpatient setting, to patients outside of the hospital, thus highlighting a role for pharmacist involvement. In this article, we will discuss, based on a review of the published literature, the important and expanding role of clinic- and home-based services, such as those provided in the patient’s home (which will be referred to as HITH) and OPAT models of care, the types of medication-based activities performed and the reported roles of pharmacists in the delivery of these types of services. Through review of the services provided, the key roles that pharmacists play and could play in these models of care will be discussed.

## 2. Why Have Clinic- and Hospital-Based Home Care, Such as HITH and OPAT?

The proportion of Australian residents aged over 65 years is expected to increase to almost 22.5% of the population by 2050 [6], and in the USA, the population of this same age group is expected to double in size between 2010 and 2050 and reach a total of 89 million people [7]. Similarly, the population growth of British residents 65 years and over increased by 22.9% to 12.4 million people in the ten years between mid-2009 and mid-2019 [8]. As chronic disease is more prevalent in aging populations, there is an increasing pressure on hospitals to provide care.

There is also an increasing trend to treat other patient groups, such as acutely or chronically ill pediatric patients, at home. Due to medical and technical advances leading to increased pediatric patient survival, the number of acutely unwell pediatric patients requiring hospital treatment has increased in recent years, and international guidelines encourage the treatment of children at home to support the patient and their family wherever possible. Therefore, not surprisingly, the number of children with serious illnesses being treated at home has recently increased [9,10].

Providing hospital-equivalent care to patients outside the hospital setting is therefore an important consideration for governments and policy makers for healthcare in the future. HITH and OPAT programs are safe [11,12,13], cost-effective [13,14,15,16] and result in positive patient outcomes [13,15,17,18]. Most patients also report a preference for HITH treatment compared to an inpatient hospital stay [13,19,20,21,22]. Patients are able to resume work and normal activities sooner, older patients experience less delirium, bowel and urinary complications [19,23,24,25,26] and both pediatric and nursing home patients experience less anxiety and disruption to their normal living environment [24,27,28]. Patients treated at home also have less exposure to hospital-acquired infections [28].

## 3. Clinic- and Hospital-Based Home Care Models

Clinic- and hospital-based home care models differ not only between countries but also within countries. Models of care vary widely in the types of patients, medical review and involvement, treatments offered, staffing mix and location where treatment is provided. However, published studies that review and compare these various models of care with patient outcomes are lacking.

### 3.1. Hospital in the Home (HITH)

HITH services are commonly led by nursing or medical staff, with medical reviews provided either in a hospital clinic, usually at least once weekly, or in the patient’s home [29]. Physicians have varying input and frequency of review depending on the location of the HITH service. Some medical officers are employed and work within the HITH service full-time and provide daily home visits and 24-h medical support, whereas other HITH services describe limited physician assistance, often from hospital doctors affiliated with inpatient units or the emergency department, in the form of a once-weekly patient review performed in a hospital clinic [29,30]. Occasionally, medical support from within the hospital or emergency department is also required for HITH patients after hours or between medical reviews, particularly when adverse reactions or problems arise [29].

In the USA, the ‘Johns Hopkins Hospital-at-Home’ model treats patients requiring hospital-level care at home at numerous sites across the USA, whilst still considering the patient as an inpatient, and medical responsibility is maintained by the hospital [31]. Whilst the Hospital at Home physician visits the patient at home at least once daily, the Hospital at Home nurse visits the patients frequently when required and also provides 24-h support, as a substitute for acute inpatient hospital care. Eligible patients usually live within a 30-min travel time from the hospital. Patients utilizing this model are most commonly treated for community-acquired pneumonia, chronic heart failure, chronic obstructive pulmonary disease (COPD) and cellulitis [26,31].

A recently described (2017) British HITH service in the UK is ‘The Guy’s and St Thomas’ NHS Foundation Trust (GSTT) @home service’, which accepts patients from general practitioners (GPs) or via hospital referrals [32]. The service is led by a senior nurse, with consultant geriatrician support, who visits the patient at home or in the hospital outpatient clinic as required. A multidisciplinary team consisting of senior nurses, GPs, rehabilitation support workers, physiotherapists, occupational therapists, social workers and a pharmacist meet daily to discuss appropriate initial and ongoing treatment. Patients treated via this service include those with cellulitis, falls, COPD, dehydration, gastroenteritis, heart failure, infected foot ulcers, post-operative surgery, urinary tract infection, unstable diabetes, palliative care, community-acquired pneumonia, deep vein thrombosis, hyperemesis gravidarum, pyelonephritis and viral illness. Treatments include rapid assessment, diagnosis, treatment and evaluation, medication titration, intravenous/subcutaneous fluids, intravenous antibiotics, intravenous diuretics, treatment for respiratory disorders (for example, nebulizers, antibiotics and physiotherapy), bladder scans for post-gynecological surgery and trial without catheter (post-operatively) [32].

An Australian HITH service offered by the Royal Melbourne Hospital provides services to patients at home, including patients that also reside in nursing homes. Patients are considered hospital inpatients and the hospital maintains medical responsibility throughout their HITH stay [33]. Medical care is provided seven days a week and 24 h a day, and medical and nursing staff visit the patient in the nursing home or at home for medical review once or twice a day. The service administers oxygen, intravenous antibiotics and intravenous fluids as well as providing mobile pathology and radiology services [33]. Another Australian HITH service located in Western Sydney treats patients with intravenous antibiotics, manages wound care and administers injectable anticoagulants. The service is led by Infectious Disease (ID) specialists who review patients weekly in a hospital clinic. Support is provided by nursing staff and a dedicated pharmacist. Patient referrals are accepted from the Emergency Department, hospital inpatient wards or other public hospitals for patients living in the local area [30].

Other commonly described treatments provided to patients in HITH programs include the administration of intravenous antibiotics, blood transfusions, intravenous fluids, chemotherapy, anticoagulation for venous thromboembolism and perioperative bridging and post-surgical care, such as complicated wound management [4,29]. Conditions commonly treated via HITH programs include infectious diseases, venous thromboembolism, cancer, COPD, asthma exacerbations, heart failure and dehydration [4,26,34]. Researchers have recently discussed the scope for continued expansion into therapeutic areas, such as cancer and heart failure, and any other suitable areas where hospital treatment may be safely and effectively provided at home [4].

Medicines administered via the parenteral route, such as subcutaneous and intramuscular injection, as well as via infusion are all suitable for administration via clinic- and hospital-based home care services. The treatment of gastrointestinal diseases, dehydration, congestive heart failure, cancer, multiple sclerosis, rheumatoid arthritis, hemophilia, immune deficiencies and neurological disorders, which may be treated with treatments including antibiotics, antifungals, antivirals, chemotherapy, hydration therapy, pain management, parenteral nutrition, immunoglobulins and blood factors, corticosteroids, erythropoietin, monoclonal antibodies (such as infliximab or natalizumab), inotropic cardiac medications and growth hormones, have all been described in the literature [35,36,37]. Complex home infusions are also increasingly used, and an Italian study described a team of nurses and doctors that successfully administered enzyme therapy on a fortnightly basis to patients with Fabry disease at home [38]. A total of 85 Italian patients (45 males, 40 females) from 11 Italian regions successfully received a cumulative number of 4269 home infusions of agalsidase alfa with a 100% compliance rate for treatment.

### 3.2. OPAT Models of Care

OPAT is defined as the administration of parenteral, most commonly intravenous, antimicrobial therapy to patients in an outpatient setting (OPAT) for at least 2 days or more, which is common practice in clinic- and hospital-based home care. Recently published guidelines in the UK and the USA have described the popularity and expected growth of OPAT [39,40,41].

#### 3.2.1. Home-Based OPAT

As reported by the National Home Infusion Foundation in the USA in 2017, 3.2 million patients in America receive infusions at home annually, the most common of which were for infectious diseases. This represents a substantial increase from 2008, where only 829,000 patients received infusions in home-based care [13,35,41].

Home-based OPAT is described using varying models of care (Table 1). The patient or their carer can be trained to self-administer their intravenous antibiotics with ongoing oversight by a nurse who visits the patient at home at least once per week (commonly termed ‘Home Infusion’ in the USA) [40]. Alternatively, the patient self-administers OPAT at home (S-OPAT) but visits a physician’s office or clinic once to twice weekly for supervision and ongoing supplies [13,40,42]. An example of a self-administered OPAT model was described in the literature in 2015, where Subedi et al. showed the safety and efficacy of a self-administered OPAT model [42]. OPAT was administered to patients through the ‘Hospital Alternate Site Infusion Service (ASIS)’ from the Princess Alexandra Hospital in Brisbane, Australia. Patients were taught how to administer their own OPAT before they were discharged home, with ID specialists reviewing patients once weekly in a hospital clinic [42].

A home-based OPAT model commonly used in the USA by Veterans Affairs programs, as well as in Australia and Europe, is the HITH model, where a nurse visits and administers all OPAT in the patient’s home [40]. Interestingly, a recent review (2020) of Australian HITH services observed that the treatment of infectious diseases is one of the most common indications for HITH patients and this practice is expected to grow [4]. Typically, the types of infectious diseases treated within HITH programs with OPAT include treatment with both short- or longer-term intravenous therapy for indications, including cellulitis and soft tissue infections, osteomyelitis, septic arthritis, respiratory infections, such as exacerbation of bronchiectasis and cystic fibrosis, urinary tract infections and endocarditis.

#### 3.2.2. Clinic-Based OPAT

An alternate setting to HITH or self-administration models for the provision of OPAT is via a clinic, an infusion center or a skilled nursing facility (Table 1). In these models, antibiotic therapy is administered to the patient by trained staff in a physician’s office or a freestanding infusion center or the patient is admitted to a skilled nursing facility (SNF), which is more commonly used in the USA for patients who have additional nursing needs or no home infusion insurance benefits. These modes of delivery are generally used for patients unable to self-administer the therapy themselves. The nurses within skilled nursing facilities administer infusions in addition to any other services, such as physical therapy and wound care, until the patient is well enough to return home [40]. OPAT has also been reported to be administered in dialysis units in the USA [40].

## 4. Challenges in Providing Medication Services in Clinic- and Hospital-Based Home Care

### 4.1. Frequency of Service

Many of the documented challenges associated with treating OPAT patients in the home or clinic environment relate to medication administration. Nursing staff typically visit patients once to twice a day to administer treatments, or alternatively, patients visit the clinic or self-administer their OPAT treatment once or twice a day. Therefore, the administration of the prescribed treatment, such as intravenous antibiotics, often needs to be adjusted to suit this frequency. It is generally impractical for nursing staff to visit a patient at home three or four times a day, and although patients may be educated on how to self-administer their treatment, dosing multiple times a day may be too difficult or disruptive to their daily activities [43]. Therefore, if antibiotics require frequent administration, an alternative antimicrobial agent is identified or 12- to 24-h continuous intravenous infusions are commonly used. This enables administration once to twice per day to coincide with the corresponding nursing visit.

### 4.2. Antibiotic Stability and Ambulatory Intravenous Infusion Pumps

Ambulatory intravenous infusion pumps are generally used to give patients mobility in the home and community. Intravenous delivery devices, such as elastomeric pump devices or battery-operated intravenous pumps, are used to administer intravenous solutions of antibiotics continuously over prolonged periods, such as over 12 or 24 h. Whilst battery-operated pumps use batteries to power the intravenous infusion, elastomeric pump devices administer infusions using both flow restrictors in the infusion line as well as pressure applied by an internal pre-filled balloon reservoir. Antibiotic solutions administered via ambulatory infusion pumps are maintained at room temperature throughout the infusion time and, therefore, must be stable in solution at expected room temperatures to ensure the appropriate dose is administered. Published research on antibiotic stability at higher or lower room temperatures is lacking, which is particularly problematic for antibiotics that rapidly degrade at higher temperatures or for antibiotics with possibly harmful degradation products [44]. Administration rates of intravenous solutions from ambulatory intravenous pump devices must be considered since there is some evidence to suggest varying flow rates occur from elastomeric pump devices, depending on ambient temperature and viscosity of the infusion solution [45].

### 4.3. Patient Monitoring, Communication and Collaboration

Another challenge for clinic- and hospital-based home care models is the monitoring and management of patients and their treatment in a home or community setting, where there is less supervision compared to the traditional inpatient hospital environment.

Despite its many benefits, adverse reactions to OPAT are common. A recent USA study in 2017 reviewed the frequency and types of adverse drug reactions experienced in patients discharged with OPAT over a 6-month period [46]. The retrospective chart analysis found that 44.4% of patients (*n* = 144) experienced an adverse drug reaction to OPAT therapy. Of these adverse drug reactions, 26% of cases required a change in drug therapy, and a hospital readmission occurred in 9% of cases. This highlights the need for adequate monitoring and management of patients, particularly through transitions of care.

It is also recognized that patients initially accepted to HITH and OPAT services may be less clinically stable than those accepted to other types of community care services, and therefore, careful patient selection is important. This includes ensuring an initial clinical review and ongoing medical oversight by a medical officer, a stable patient condition, a suitable home environment located a reasonable distance from the service and adequate carer support at home [39]. Clear guidelines and patient review by ID physicians, pharmacists and nurses before the patient is accepted, as well as regular and ongoing reviews while the patient is at home, are essential to ensure patients receive the appropriate level of care, monitoring and treatment at home [39].

Geographical separation between patient and healthcare staff is a potential barrier to effective communication and, therefore, optimum patient care. This is particularly important if there is patient deterioration and the referral for appropriate and timely management by HITH and OPAT staff. Clearly written policies and procedures and adequate ongoing handover of patient care via communication and appropriate documentation are essential to overcome this challenge. Patients should be educated about possible signs and symptoms or adverse effects to their medication therapy and encouraged to contact staff immediately with any questions or concerns [39].

### 4.4. Antimicrobial Stewardship in OPAT Services

Antimicrobial stewardship (AMS) is a multidisciplinary and collaborative process that aims to reduce antibiotic resistance in the long term by promoting the effective and appropriate use of antibiotics in the short term. Access to antimicrobial stewardship (AMS) programs and specialist staff, such as ID specialists, may be a challenge for some clinic- and home-based services providing OPAT services.

A recent survey reviewing the role and effectiveness of antimicrobial stewardship (AMS) in Australian HITH/OPAT services reported that not all Australian HITH/OPAT services had the same access to AMS programs [47]. The same survey also indicated that AMS interventions within these OPAT services could be improved in the future.

Recent research into OPAT models of care have demonstrated the importance of ID physician involvement, including the benefits of an ID specialist review before the patient is discharged from the hospital with OPAT, resulting in a reduction in unnecessary therapy and ensuring appropriate ongoing monitoring and review [48]. British and American OPAT guidelines also recommend the inclusion of ID physicians in OPAT services to ensure optimum outcomes [39,40]. Disappointingly, however, a recent American survey of ID physicians reported that 60% of institutions providing OPAT services did not require a mandatory ID review before the patient was discharged [49]. This practice was not supported by 75% of the ID specialists surveyed, concluding that ID involvement in OPAT programs could be improved. A successful pharmacist-led OPAT model reported in 2016 described a pharmacist working collaboratively with ID physicians, suggesting an important opportunity for pharmacists to further assist ID specialists and AMS stewardship activities in the OPAT setting [50].

## 5. Pharmacist Involvement in Clinic- and Hospital-Based Home Care

Many of the treatments administered in clinic- and hospital-based home care programs, such as intravenous antibiotics, chemotherapy and anticoagulants, are classified as high-risk medications, where supply and clinical review by a clinical pharmacist is recommended. The ‘High-Risk Medication Policy’ published by the Clinical Excellence Commission in Australia in 2019 [51] identifies these treatments as having a high potential for medication-related harm. The policy outlines the importance of a pharmaceutical review for all patients treated with high-risk medications and suggests it should be ‘conducted or supervised by a qualified and suitably trained health professional (ideally a pharmacist) acting as part of a multidisciplinary team’ [51].

The type and extent of pharmacist involvement in clinic- and home-based hospital care described in the literature varies considerably. An Australian review of pharmacy services provided to HITH ranged from a dedicated HITH pharmacist to no pharmacist involvement, other than the dispensing and supply of medications [52]. For the HITH services that have a dedicated pharmacist, service provision includes aseptic manufacture; provision of drug stability, storage and intravenous pump information; therapeutic drug monitoring and dosing advice; patient counselling and administrative services, such as policy and procedure development.

## 6. Benefits of Pharmacist Involvement

Pharmacists can significantly improve patient care in clinic- and hospital-based home care, as highlighted in published studies. Most notably, pharmacists have demonstrated positive contributions in the areas of antimicrobial stewardship, clinically monitoring and managing patients on OPAT and clinical medication reviews at transitions of care.

### 6.1. Appropriate Prescribing of Antimicrobials (AMS)

With a large portion of patients treated for infectious diseases outside the hospital setting, and the threat of antimicrobial resistance, pharmacists in the HITH/OPAT setting are in an excellent position to provide evidence-based guidance on the safe and effective administration of intravenous antibiotics to patients in HITH and OPAT programs. The World Health Organisation lists antibiotic resistance as ‘one of the biggest threats to global health, food security and development today’, and pharmacists, in partnership with ID specialists, can effectively encourage the appropriate use of antibiotics in HITH programs [39]. Pharmacists can ensure that the most appropriate antibiotic is prescribed and administered in a safe and effective manner via infusion devices, if required, at the most appropriate dose, formulation and time frame, with a timely intravenous to oral switch and cessation of therapy when appropriate, in addition to reviewing and reporting patient outcomes, healthcare-associated acquired infections and re-admission rates [53].

Research conducted in the Royal Children’s Hospital HITH service in Melbourne, Australia, in 2012/2013 highlighted the need for a dedicated pharmacist [54]. This 12-month prospective observational study reviewed the use, appropriateness, and outcomes of OPAT in children. The lack of a dedicated pharmacist was listed as a possible cause for the noted deviation from hospital antibiotic policies, particularly for issues such as incorrect dose or duration of antibiotic.

Heintz et al. showed that pharmacists within a multidisciplinary team consisting of ID specialists and nurses can help to improve the safety, efficacy and convenience of OPAT as well as reduce costs when OPAT treatment is reviewed prior to the patient being discharged from hospital [55]. Within the prospective analysis over a 12-month period, the pharmacist’s interventions positively impacted the safety, regimen complexity and efficacy of OPAT treatment. The multidisciplinary team also prevented unnecessary delays to hospital discharge and avoided unnecessary OPAT treatment (where no antibiotic was required or an oral antibiotic was indicated, for example), which led to substantial cost savings for the service.

An initial OPAT patient review and ongoing reviews by a pharmacist were described by Chung et al. (2016) in a pharmacist-managed OPAT program within a county teaching hospital in Indianapolis, Indiana [50]. Working collaboratively with ID physicians, the pharmacist advised on appropriate patient and regimen selection, reviewed and confirmed OPAT orders when the patient was discharged from hospital and ensured ongoing laboratory monitoring was performed. The pharmacist also monitored the patient for any adverse effects and line complications and organized the removal of the vascular access device or pump at completion of the treatment. The pharmacist-led OPAT program demonstrated improvements in patient outcomes with minimal adverse effects and line complications.

### 6.2. Patient Monitoring, Clinical Review and Adverse Effects

A significant activity for pharmacists working within a multidisciplinary OPAT team is the initial and ongoing clinical review of the patient. This includes identifying and reviewing the clinical pathology and therapeutic drug monitoring, monitoring for potential medication-related adverse effects and assessing the patient response to treatment.

The ‘American Society of Health-System Pharmacists (ASHP) Guidelines on Home Infusion Pharmacy Services’ in the USA identified that pharmacists should participate in initial HITH or OPAT patient clinical reviews, including reviewing patient pathology results, renal function, medication interactions, patient allergies and other clinically relevant patient information, before supplying any new medication or ongoing medication [56]. Recently published British OPAT guidelines recommended that patients receiving OPAT have pathology testing at least once per week, including a full blood count, renal and liver function, C-reactive protein (CRP) and therapeutic drug monitoring, if indicated, as well as other tests if specified for specific indications or medication therapies [39]. Pharmacists are in an ideal position to ensure that appropriate clinical monitoring occurs and is reviewed when supplying parenteral antimicrobial medications to patients in OPAT programs, ensuring safe and effective patient care.

Shah et al. highlighted that non-ID physicians at an infusion center are less successful at following OPAT laboratory monitoring guidelines compared to ID physicians [57]. However, the addition and assistance of a pharmacist improved the rates and appropriateness of laboratory monitoring compliance for the non-ID physician group, and the researchers concluded that pharmacist assistance may be especially important in facilities without ID oversight, as pharmacists may be more readily available.

### 6.3. Medication Review

Medication errors relating to transitions of care, as well as frequent medication-related hospital admissions, are well described [58]. Pharmacists have the required skills to ensure that medications are appropriately managed in the home setting, especially following transitions of care, via medication history documentation, medication reconciliation and clinical review. Pharmacists successfully provide medication reviews at home visits, in hospital clinics, via telehealth or via telephone calls, as demonstrated by recent studies outlining pharmacist activities in this area [59,60,61,62].

Corsi et al. reviewed the impact of a pharmacist medication review service in a visiting nurse service (VNS) in 2017 [63]. An average of 8.7 medication-related problems per patient were identified and reviewed by the pharmacist, including immunization requirement, improving adherence (inappropriate administration or technique or overuse), dose adjustment, adverse drug reaction, missing drug therapy, suboptimal drugs, cost-effective alternative, unnecessary treatment and drug interaction. Cost-saving benefits of these pharmacist interventions included prevention of hospital or ED admission, reducing medication cost, preventing life-threatening situations, adherence support, preventing physician visits and preventing additional physician orders. Following completion of the study, a full-time pharmacist position for the service was created.

In 2011, an Australian study in Melbourne described the success of a pharmacist-led anticoagulant dosing service within an Australian HITH program [64]. Patients achieved a therapeutic INR faster when warfarin dosing was managed by a pharmacist compared to a doctor.

In 2019, a medication reconciliation and review service was provided by a pharmacist at a home visit, with a view to reduce cholinergic burden, for patients suffering from delirium within a Hospital in the Home program [25].

Working within the patient’s home and the outpatient or clinic environment also places the pharmacist in an ideal position to integrate into the patient-centered medical home. Linking in with the patient, the patient’s general practitioner and the relevant hospital medical staff allows pharmacists to assist with the coordination of specialized treatments between hospital and community whilst also being readily available to the patient and ensuring the patient’s needs are appropriately met [65].

### 6.4. Specialised OPAT Medication Supply

Accurate and timely medication supply is an important role for pharmacists to ensure continuity of care for patients. The supply of parenteral medications, such as intravenous antibiotics administered via ambulatory pumps, requires the ordering, manufacture and/or dispensing of aseptically prepared antibiotic solutions. Such products often have a short shelf-life and require refrigeration during storage. Therefore, frequent communication between pharmacists and prescribing doctors, OPAT nurses and the patient/carer is required to ensure seamless ongoing treatment. The pharmacist can prevent disruptions to treatment by responding promptly to therapy changes and, therefore, reduce unnecessary medication wastage costs. Examples of therapy changes include dose changes following changes in renal function or a change in antibiotic if a patient is not responding or experiences adverse effects.

The ‘American Society of Health-System Pharmacists (ASHP) Guidelines on Home Infusion Pharmacy Services’ outline the role of pharmacists providing intravenous infusions for patients in the home setting [56]. These guidelines state that pharmacist activities fall into three broad areas: ‘the provision of specialized, complex pharmaceutical products; development and execution of plans to manage the medication therapy of patients; and clinical assessment and monitoring of patients in their homes’. Specifically, the role of the pharmacist as outlined by these guidelines should include duties such as supervision of dispensing and delivery of sterile preparations; drug information to colleagues and patients; clinical monitoring and ongoing clinical review of patients at home on infusions; effective use of technology to ensure work efficiency and patient safety; maintaining patient confidentiality and using support staff effectively.

### 6.5. Specialized Advice, Patient Education and Quality Improvement

Pharmacists are an excellent source of specialist knowledge within the multidisciplinary team. In the use of antibiotic administration via ambulatory infusion pumps, they provide expertise on antibiotic stability factors (including review of diluent, concentration, infusion container or different pump types), storage conditions (room and refrigerator temperature), infusion time and treatment costs. The pH and osmolality of solutions may also need to be reviewed by the pharmacist, depending on the type of vascular access device used [40]. Practical advice concerning the use of parenteral medications outside the hospital setting and overcoming challenges faced by limited nursing or clinic visits and long intravenous infusion times are increasingly important considerations, especially as new research emerges and evidence and advice is updated.

Without definitive stability data for all intravenous antibiotics used in continuous infusions, it is expected that treatments may vary between clinic- and home-based hospital models of care depending on local policy and decision-making. These factors are described in an early Australian study describing the activities of HITH pharmacists in Victoria, where a range of practices were observed relating to drug selection and dosing regimens for HITH patients [52]. Due to the rapidly evolving nature of HITH and OPAT, it is important for pharmacists to be conscious of emerging research, particularly in the field of antibiotic stability, to support evidence-based practice. There is potential to standardize some aspects of patient care relating to choice and administration of intravenous antibiotics in HITH programs. Assisting with policy and practice development and educating patients and other health professionals are also important roles of the pharmacist as hospital care is increasingly provided outside the hospital setting. Pharmacists should also be involved in all aspects of service improvement, benchmarking and documenting patient outcomes [39].

## 7. Conclusions

With an ageing population, providing hospital-associated patient care in a clinic- and hospital-based home care setting instead of an inpatient hospital setting has many benefits. This includes increasing hospital capacity without increasing costs or adversely affecting patient outcomes. Patients mostly prefer to be treated at home and can resume normal activities sooner. Pediatric patients, especially those that are very unwell with ongoing complex needs, benefit immensely from clinic- and home-based treatment models. Avoiding a stressful hospital stay has a profoundly positive impact on the well-being of the child, and these models also ensure that parents can be more involved in their child’s overall care.

However, with the growth of these models of care, it is important that appropriate resources and staff are provided to support good-quality, patient-focused care. Pharmacists have demonstrated a significantly positive effect on improving the efficacy, safety and favorable outcomes in HITH models, particularly within OPAT services, and can also help to address many of the identified challenges in providing clinic- and hospital-based home care. Pharmacists have an opportunity to extend their roles in these settings beyond the traditional supply role, including providing expert clinical advice to patients and colleagues, providing specialist advice on the practical use of antimicrobials and other parenteral medications administered in the community and providing medication reviews at transitions of care. Participating in continuing education, quality improvement and research is also important for pharmacists to ensure evidence-based practice is encouraged within clinic- and hospital-based home care models.

Pharmacists are, or should be, an integral part of HITH and OPAT multidisciplinary teams and their role should continue to evolve as clinic- and hospital-based home care continues to expand and grow into new areas of service in the future. The integration and expansion of the roles of pharmacists in these models of care would be assisted by future studies which focus on health economic analyses to demonstrate benefits arising from the differing models of care and patient outcomes.

## Figures and Tables

**Table 1:** Summary of models of clinic- and hospital-based home care for outpatient parenteral antimicrobial therapy (OPAT) administration [42,43].

Home-Based	Home infusion: Patient or carer self-administers OPAT with oversight by home infusion nurse
	Clinic: Patient self-administers OPAT at home with reviews by a physician in the physician’s office or a clinic twice weekly
	Hospital in the Home: nurse administers OPAT in the home with physician reviews either at home or in the clinic
Clinic-Based	Infusion center-based or clinic-based: OPAT administered by trained nurses in a physician’s office or a freestanding infusion center
	Skilled nursing facility: OPAT administered by trained nurses to patients with additional nursing needs. Rehabilitation wards/services or nursing homes may be used. Patients stay overnight until they are well enough to return home

## References

[1] Black I. (1953). Hospital Home Care. Can. J. Public Health (Rev. Can. de Sante’e Publique).

[2] Terrade O.T.T., Cottencin A. (2012). Hospital care at home in France: An alternative to conventional hospitalization with the same obligations towards quality and administration. J. Natl. Inst. Health.

[3] Victoria Health (2011). Australia. Hospital in the Home Guideline. www.health.vic.gov.au/hith/.

[4] Montalto M., McElduff P., Hardy K. (2020). Home ward bound: Features of hospital in the home use by major Australian hospitals, 2011–2017. Med. J. Aust..

[5] Rucker R.W., Harrison G.M. (1974). Outpatient intravenous medications in the management of cystic fibrosis. Pediatrics.

[6] McPake B., Mahal A. (2017). Addressing the Needs of an Aging Population in the Health System: The Australian Case. Health Syst. Reform.

[7] Dall T.M., Gallo P.D., Chakrabarti R., West T., Semilla A.P., Storm M.V. (2013). An Aging Population and Growing Disease Burden Will Require ALarge and Specialized Health Care Workforce By 2025. Health Aff..

[8] Office for National Statistics (2019). Population Estimates for the UK, England & Wales, Scotland & Northern Ireland: Mid. https://www.ons.gov.uk/peoplepopulationandcommunity/populationandmigration/populationestimates/bulletins/annualmidyearpopulationestimates/mid2019estimates.

[9] Ward C., Glass N., Ford R. (2014). Care in the home for seriously ill children with complex needs: A narrative literature review. J. Child. Health Care.

[10] Hansson H., Brødsgaard A. (2016). Effectiveness and safety of intravenous therapy at home for children and adolescents with acute and chronic illnesses. JBI Database Syst. Rev. Implement. Rep..

[11] Montalto M. (1998). How safe is hospital-in-the-home care?. Med. J. Aust..

[12] Montalto M. (1999). Hospital in the home: Take the evidence and run. Med. J. Aust..

[13] Polinski J.M., Kowal M.K., Gagnon M., Brennan T.A., Shrank W.H. (2017). Home infusion: Safe, clinically effective, patient preferred, and cost saving. Health.

[14] Board N., Brennan N., Caplan G. (2000). A randomised controlled trial of the costs of hospital as compared with hospital in the home for acute medical patients. Aust. N. Z. J. Public Health.

[15] Caplan G.A., Sulaiman N.S., Mangin D., Ricauda N.A., Wilson A.D., Barclay L. (2012). A meta-analysis of “hospital in the home”. Med. J. Aust..

[16] MacIntyre C.R., Ruth D., Ansari Z. (2002). Hospital in the home is cost saving for appropriately selected patients: A comparison with in-hospital care. Int. J. Qual. Health Care.

[17] Shepperd S., Doll H., Angus R.M., Clarke M.J., Iliffe S., Kalra L., Ricauda N.A., Tibaldi V., Wilson A.D. (2009). Avoiding hospital admission through provision of hospital care at home: A systematic review and meta-analysis of individual patient data. Can. Med. Assoc. J..

[18] Sriskandarajah S., Hobbs J.G., Roughead E.E., Ryan M.K., Reynolds K. (2018). Safety and effectiveness of ‘hospital in the home’ and ‘outpatient parenteral antimicrobial therapy’ in different age groups: A systematic review of observational studies. Int. J. Clin. Pract..

[19] Caplan G.A., Ward J., Brennan N.J., Coconis J., Board N., Brown A. (1999). Hospital in the home: A randomised controlled trial. Med. J. Aust..

[20] Fracgp M.M.M.B. (1996). Patients’ and Carers’ Satisfaction with Hospital-in-the-Home Care. Int. J. Qual. Health Care.

[21] Leff B., Burton L., Mader S., Naughton B., Burl J., Clark R., Greenough W.B., Guido S., Steinwachs N., Burton J.R. (2006). Satisfaction with Hospital at Home Care. J. Am. Geriatr. Soc..

[22] Facultad J., Lee G. (2019). Patient satisfaction with a hospital-in-the-home service. Br. J. Commun. Nurs..

[23] Caplan G. (2008). Does ‘Hospital in the Home’ treatment prevent delirium?. Aging Health.

[24] Caplan G., Coconis J., Woods J. (2005). Effect of Hospital in the Home Treatment on Physical and Cognitive Function: A Randomized Controlled Trial. J. Gerontol. Ser. A Biol. Sci. Med. Sci..

[25] Chia J., Eeles E., Tattam K., Yerkovich S.T. (2020). Outcomes for patients with delirium receiving hospital-in-the-home treatment: An Australian perspective. Australas. J. Ageing.

[26] Leff B., Burton L., Mader S.L., Naughton B., Burl J., Inouye S.K., Greenough W.B., Guido S., Langston C., Frick K.D. (2005). Hospital at Home: Feasibility and Outcomes of a Program to Provide Hospital-Level Care at Home for Acutely Ill Older Patients. Ann. Intern. Med..

[27] Balaguer A., De Dios J.G. (2015). Home versus hospital intravenous antibiotic therapy for cystic fibrosis. Cochrane Database Syst. Rev..

[28] Patel S., Abrahamson E., Goldring S., Green H., Wickens H., Laundy M. (2014). Good practice recommendations for paediatric outpatient parenteral antibiotic therapy (p-OPAT) in the UK: A consensus statement. J. Antimicrob. Chemother..

[29] Montalto M. (2010). The 500-bed hospital that isn’t there: The Victorian Department of Health review of the Hospital in the Home program. Med. J. Aust..

[30] Li W., Branley J., Sud A. (2018). Outpatient parenteral antibiotic therapy in a suburban tertiary referral centre in Australia over 10 years. Infection.

[31] Leff B. (2009). Defining and disseminating the hospital-at-home model. Can. Med. Assoc. J..

[32] Lee G.A., Titchener K. (2016). The Guy’s and St Thomas’s NHS Foundation Trust @home service: An overview of a new service. Lond. J. Prim. Care.

[33] Montalto M., Chu M.Y., Ratnam I., Spelman T., Thursky K. (2015). The treatment of nursing home-acquired pneumonia using a medically intensive Hospital in the Home service. Med. J. Aust..

[34] Cryer L., Shannon S.B., Van Amsterdam M., Leff B. (2012). Costs for ‘Hospital at Home’ Patients Were 19 Percent Lower, With Equal or Better Outcomes Compared to Similar Inpatients. Health Aff..

[35] National Home Infusion Association ‘About Home Infusion’. https://www.nhia.org/about-infusion-therapy/.

[36] Barfield E., Sockolow R., Hoffenberg E., Saeed S., Kim S., Siebold L., Picoraro J., Moses J., Dykes D., Grossman A. (2018). Assuring Quality for Non-hospital–based Biologic Infusions in Pediatric Inflammatory Bowel Disease: A Clinical Report from the North American Society for Pediatric Gastroenterology, Hepatology and Nutrition. J. Pediatr. Gastroenterol. Nutr..

[37] Qian W., Lam T.N., Lam H.H.W., Li C.-K., Cheung Y.T. (2019). Telehealth Interventions for Improving Self-Management in Patients with Hemophilia: Scoping Review of Clinical Studies. J. Med. Internet Res..

[38] Concolino D., Amico L., Cappellini M., Cassinerio E., Conti M., Donati M., Falvo F., Fiumara A., Maccarone M., Manna R. (2017). Home infusion program with enzyme replacement therapy for Fabry disease: The experience of a large Italian collaborative group. Mol. Genet. Metab. Rep..

[39] Chapman A.L.N., Patel S., Horner C., Green H., Guleri A., Hedderwick S., Snape S., Statham J., Wilson E., Gilchrist M. (2019). Updated good practice recommendations for outpatient parenteral antimicrobial therapy (OPAT) in adults and children in the UK. JAC Antimicr. Resist..

[40] Norris A.H., Shrestha N.K., Allison G.M., Keller S., Bhavan K.P., Zurlo J.J., Hersh A.L., Gorski L.A., Bosso J., Rathore M.H. (2019). 2018 Infectious Diseases Society of America Clinical Practice Guideline for the Management of Outpatient Parenteral Antimicrobial Therapya. Clin. Infect. Dis..

[41] Shah A., Norris A. (2016). Handbook of Outpatient Parenteral Antmicrobial Therapy for Infectious Diseases.

[42] Subedi S., Looke D., McDougall D., Sehu M., Playford E. (2015). Supervised self-administration of outpatient parenteral antibiotic therapy: A report from a large tertiary hospital in Australia. Int. J. Infect. Dis..

[43] Steffens E., Quintens C., Derdelinckx I., Peetermans W.E., Van Eldere J., Spriet I., Schuermans A. (2018). Outpatient parenteral antimicrobial therapy and antibiotic stewardship: Opponents or teammates?. Infection.

[44] Jenkins A., Hills T., Santillo M., Gilchrist M. (2017). DI-010 A systematic literature review of antimicrobial stability data in elastomeric devices. Drug Inf. Pharmacother..

[45] Kawabata Y. (2011). Effect of coefficient of viscosity and ambient temperature on the flow rate of drug solutions in infusion pumps. Pharm. Dev. Technol..

[46] Hale C.M., Steele J.M., Seabury R.W., Miller C.D. (2017). Characterization of Drug-Related Problems Occurring in Patients Receiving Outpatient Antimicrobial Therapy. J. Pharm. Pract..

[47] Friedman N.D. (2018). 1855. Antimicrobial Stewardship (AMS) and the Outpatient Parenteral Antimicrobial Therapy (OPAT) Setting. Open Forum Infect. Dis..

[48] Conant M.M., Erdman S.M., Osterholzer D. (2014). Mandatory infectious diseases approval of outpatient parenteral antimicrobial therapy (OPAT): Clinical and economic outcomes of averted cases. J. Antimicrob. Chemother..

[49] Muldoon E.G., Switkowski K.M., Tice A., Snydman D., Allison G.M. (2014). A national survey of infectious disease practitioners on their use of outpatient parenteral antimicrobial therapy (OPAT). Infect. Dis..

[50] Chung E.K., Beeler C.B., Muloma E.W., Osterholzer D., Damer K.M., Erdman S.M. (2016). Development and implementation of a pharmacist-managed outpatient parenteral antimicrobial therapy program. Am. J. Health Pharm..

[51] Clinical Excellence Commission Australia NSW Government High Risk Medicines Management Policy PD2019_058. https://www1.health.nsw.gov.au/pds/ActivePDSDocuments/PD2019_058.pdf.

[52] Ioannides-Demos L.L. (1999). Survey of Pharmacy Services and Drug Usage in Victorian Public Hospital HITH Programs. Aust. J. Hosp. Pharm..

[53] MarkGilchrist M., Seaton R.A. (2014). Outpatient parenteral antimicrobial therapy and antimicrobial stewardship: Challenges and checklists. J. Antimicrob. Chemother..

[54] Hodgson K.A., Huynh J., Ibrahim L.F., Sacks B., Golshevsky D., Layley M., Spagnolo M., Raymundo C.-M., Bryant P.A. (2016). The use, appropriateness and outcomes of outpatient parenteral antimicrobial therapy. Arch. Dis. Child..

[55] Heintz B.H., Halilovic J., Christensen C.L. (2011). Impact of a Multidisciplinary Team Review of Potential Outpatient Parenteral Antimicrobial Therapy Prior to Discharge from an Academic Medical Center. Ann. Pharmacother..

[56] Petroff B.J., Filibeck D., Nowobilski-Vasilios A., Olsen R.S., Rollins C.J., Johnson C. (2014). ASHP Guidelines on Home Infusion Pharmacy Services. Am. J. Health Pharm..

[57] Shah P.J., Bergman S.J., Graham D.R., Glenn S. (2014). Monitoring of Outpatient Parenteral Antimicrobial Therapy and Implementation of Clinical Pharmacy Services at a Community Hospital Infusion Unit. J. Pharm. Pract..

[58] Rougheadl S.S., Rosenfeld E. Australian Commission on Safety and Quality in Healthcare Literature Review: Medication Safety Australia 2013. https://www.safetyandquality.gov.au/sites/default/files/migrated/Literature-Review-Medication-Safety-in-Australia-2013.pdf.

[59] Delate T., Chester E., Stubbings T.W., Barnes C. (2008). Clinical Outcomes of a Home-Based Medication Reconciliation Program after Discharge from a Skilled Nursing Facility. Pharmacother. J. Hum. Pharmacol. Drug Ther..

[60] Gudi S.K., Kashyap A., Chhabra M., Rashid M., Tiwari K.K. (2019). Impact of pharmacist-led home medicines review services on drug-related problems among the elderly population: A systematic review. Epidemiol. Health.

[61] Pherson E., Shermock K.M., Efird L.E., Gilmore V.T., Nesbit T., Leblanc Y., Brotman D.J., Deutschendorf A., Swarthout M.D. (2014). Development and implementation of a postdischarge home-based medication management service. Am. J. Health Pharm..

[62] Hanjani L.S., Caffery L.J., Freeman C., Peeters G., Peel N.M. (2020). A scoping review of the use and impact of telehealth medication reviews. Res. Soc. Adm. Pharm..

[63] Corsi K., Lemay V., Orr K.K., Cohen L. (2018). Pharmacist medication therapy management in home health care: Investigation of a sustainable practice model. J. Am. Pharm. Assoc..

[64] Dooley M.J., McGuiness J.V., Choo S., Ngo-Thai L.-L., Tong E., Neave K., Poole S., Street A. (2011). Successful Implementation of a Pharmacist Anticoagulant Dosing Service in Ambulatory Care. J. Pharm. Pract. Res..

[65] Agency for Clinical Innovation Key Principles of Patient Centred Medical Homes. https://www.aci.health.nsw.gov.au/nhn/patient-centred-medical-home-model/key-principles.

